# Editorial: Molecular determinants of protein assemblies in health and disease, Volume II

**DOI:** 10.3389/fmolb.2023.1343082

**Published:** 2023-12-11

**Authors:** Verena Kohler, Anoop Arunagiri, Salvador Ventura, Sonja Kroschwald, Srivastav Ranganathan

**Affiliations:** ^1^ Department of Molecular Biology, Faculty of Medicine, Umeå University, Umeå, Sweden; ^2^ Department of Internal Medicine, University of Michigan, Ann Arbor, MI, United States; ^3^ Departament de Bioquímica i Biologia Molecular, Institut de Biotecnologia i de Biomedicina (IBB), Universitat Autònoma de Barcelona, Barcelona, Spain; ^4^ Department of Biology, Institute of Biochemistry, Zürich, Switzerland; ^5^ Department of Chemistry and Chemical Biology, Harvard University, Cambridge, MA, United States

**Keywords:** protein aggregation, amyloid, RT QuIC, disaggregation, chaperones

The function of protein assemblies heavily relies on dynamic interactions among the constituent proteins or between them and other biomolecules, leading to the formation of complex structures. The focus of the Research Topic is protein–protein interactions, protein/peptide self-assembly, and the potential influencers of these events. The delicate balance governing protein self-assembly in living cells necessitates the intricate machinery of proteostasis to harness its benefits while mitigating its detrimental effects. Cellular proteostasis mechanisms play a pivotal role in orchestrating protein synthesis, folding, self-assembly, disassembly, and degradation, thereby ensuring cellular health and function. Understanding the molecular forces that underlie the production, reversibility, and turnover of dynamic biomolecular condensates and static protein aggregates is essential for appreciating how living systems meticulously balance the functional *versus* pathological features of the self-assembled entities.

This Research Topic harbors research that sheds light on the complex world of the proteostasis network, consisting of chaperones and folding factors, unraveling their fundamental mechanisms while also focusing on their implications for diseases, diagnostics, and potential treatment strategies.

Fauvet et al. extensively explored the interplay between bacterial Hsp90 and the ubiquitous Hsp70–Hsp40 axis, which holds primary responsibility for cellular protein homeostasis. Through comparative proteomic analyses of total and insoluble fractions, they found that Hsp90 functions in the mediation of the degradation of aggregation-prone Hsp70-Hsp40 substrates while working together with the proteasome HslUV. In non-stressed cells, Hsp90 appears to regulate polypeptide folding, but when stress conditions incapacitate or overwhelm the Hsp70–Hsp40 axis, Hsp90 becomes upregulated, leading to enhanced degradation of misfolded polypeptides.

Not only external stress but also internal cues involving disease-associated mutations can disrupt chaperone networks, leading to proteostasis breakdown and the emergence of toxic aggregation events. Neurodegenerative diseases, including proteinopathies like Dementia with Lewy Bodies, Alzheimer’s Disease, and Parkinson’s Disease, have a profound impact on cellular proteostasis, resulting in severe consequences, ultimately culminating in cell death. A robust and precise diagnostic process is essential but is so far missing. In this context, Peña-Bautista et al. conducted a meta-analysis on Real-time Quaking-induced Conversion (RT-QuIC) for detecting alpha-synuclein seeding activity, a central pathology in Dementia with Lewy Bodies and other synucleinopathies, including Parkinson’s Disease. Their findings demonstrated that RT-QuIC not only exhibited high diagnostic accuracy but also proved effective in diagnosing early disease stages. While it excelled in discerning samples from individuals with Dementia with Lewy Bodies from both control samples and those obtained from Alzheimer’s Disease patients, the authors noted that discrimination between different synucleinopathies was somewhat limited and found the scarcity of available studies being a limitation of their meta-analysis. Overall, RT-QuIC holds great potential as a diagnostic tool.

A substantial number of age-dependent diseases, including neurodegenerative disorders and inherited cerebrovascular conditions like CADASIL (Cerebral Autosomal Dominant Arteriopathy with Subcortical Infarcts and Leukoencephalopathy) currently lack robust and reliable treatment options. This necessitates comprehensive research into potential therapies. Oliveira et al. investigated the NOTCH3 protein, which, when mutated, misfolds and aggregates. The aggregated state is part of the material that surrounds arteries and most likely is involved in vascular smooth muscle cell degradation. This affects blood flow regulation, neuronal cell death, *etc.* Thus, it plays a pivotal role in CADASIL, the most prevalent familial form of stroke. Through *in vitro* studies involving a mutated, recombinant NOTCH3 (R133C) protein, they discovered that human BRICHOS, an ATP-independent chaperone-like protein, exhibited a potent anti-aggregating effect by stabilizing the monomeric form of mutant NOTCH3. The authors envision exploring the effect of BRICHOS on different CADASIL mutations. Also, they emphasize the promise of these *in vitro* studies and the necessity for subsequent *in vivo* investigations to unearth treatment options for CADASIL.

A critical factor in evaluating potential treatment options is minimizing or eliminating the cytotoxicity of accumulating amyloid fibrils. The importance of this was vividly demonstrated by Sulatsky et al. **,** who explored the potential consequences of different degradation strategies on two species of mature amyloid fibrils. Regardless of the degradation method employed, whether it involved protease activity, denaturants, ultrasounds, or other means, the resulting fragments retained some amyloid-like properties. Notably, in certain cases, cytotoxicity appeared to be higher than that of intact amyloids. This underscores the need for caution and an awareness of unforeseen outcomes related to incomplete amyloid degradation.

In summary, this Research Topic presents a wide spectrum of intriguing findings, spanning fundamental work on chaperone functionality to the meta-analysis of a novel diagnostic approach and compelling treatment strategies, all the while highlighting potential risks associated with these endeavors.

